# Parenting Stress Related to Behavioral Problems and Disease Severity in Children with Problematic Severe Asthma

**DOI:** 10.1007/s10880-015-9423-x

**Published:** 2015-06-09

**Authors:** Marieke Verkleij, Erik-Jonas van de Griendt, Vivian Colland, Nancy van Loey, Anita Beelen, Rinie Geenen

**Affiliations:** Merem Netherlands Asthma Center, Davos, Switzerland; Merem Asthma Center Heideheuvel, Hilversum, The Netherlands; Department of Pediatric Psychology, VU University Medical Center, Reception L, PO Box 7057, 1007 MB Amsterdam, The Netherlands; DeKinderkliniek and Flevo Hospital, Almere, The Netherlands; Department of Pediatric Psychology, Emma Children’s Hospital Academic Medical Center, Amsterdam, The Netherlands; Department of Clinical & Health Psychology, Utrecht University, Utrecht, The Netherlands; Association of Dutch Burns Centers, Beverwijk, The Netherlands; Department of Rehabilitation, Academic Medical Center, Amsterdam, The Netherlands; Department of Rheumatology & Clinical Immunology, University Medical Center Utrecht, Utrecht, The Netherlands

**Keywords:** Asthma, Behavioral problems, Child, Parenting stress

## Abstract

Our study examined parenting stress and its association with behavioral problems and disease severity in children with problematic severe asthma. Research participants were 93 children (mean age 13.4 ± 2.7 years) and their parents (86 mothers, 59 fathers). As compared to reference groups analyzed in previous research, scores on the Parenting Stress Index in mothers and fathers of the children with problematic severe asthma were low. Higher parenting stress was associated with higher levels of internalizing and externalizing behavioral problems in children (Child Behavior Checklist). Higher parenting stress in mothers was also associated with higher airway inflammation (FeNO). Thus, although parenting stress was suggested to be low in this group, higher parenting stress, especially in the mother, is associated with more airway inflammation and greater child behavioral problems. This indicates the importance of focusing care in this group on all possible sources of problems, i.e., disease exacerbations and behavioral problems in the child as well as parenting stress.

## Introduction

Asthma, a chronic inflammatory disease of the airways, is common in children and adolescents with a reported worldwide prevalence ranging from 5 to 15 % (Anandan, Nurmatov, van Schayck, & Sheikh, 2010). A small portion of pediatric asthma patients (the precise prevalence is unknown) has problematic severe asthma, defined as asthma that is not under control despite optimal treatment (Hedlin et al., 2010). Psychosocial factors may influence disease progression and the psychological status of children with asthma. The focus of this article is on parenting stress in parents of children with problematic severe asthma. Parenting stress is both a potential cause and consequence of the disease status and behavioral problems in children with problematic severe asthma.

Open (family) systems models such as the BioBehavioral Family Model (Wood, Klebba, & Miller, 2000; Wood et al., 2008) emphasize the interplay between physiological vulnerability, the family and involvement of the child in parental conflict in relation to symptom severity. Parenting stress can exert an influence on the disease through poor treatment adherence (Klok, Lubbers, Kaptein, & Brand, 2014), or through physiological stress response systems such as the sympathetic nervous system and the hypothalamic pituitary adrenal axis (Miller, Wood, Lim, Ballow, & Hsu, 2009). In support of the biobehavioral family model, in asthmatic children, the child’s perception of parental conflict showed trends of association with insecure father-child relatedness and triangulation (indirect communication of one family member with another through a third family member (Wood et al. 2000). Moreover, it was observed that these psychosocial systemic variables were associated with respiratory sinus arrhythmia, a measure of vagal activation that may underlie airway obstruction in asthma. Another study showed that children with asthma who simultaneously experienced acute and chronic stress exhibited a 5.5-fold reduction in glucocorticoid receptor mRNA and a 9.5-fold reduction in beta (2)-adrenergic receptor mRNA relative to children with asthma without comparable stressor exposure (Miller & Chen, 2006). Thus, stress was associated with physiological factors that play a role in asthma control. To the extent that reduction in glucocorticoid receptor mRNA reflects diminished sensitivity to the anti-inflammatory properties of glucocorticoids and the reduction in beta (2) adrenergic receptor mRNA reflects a reduction of bronchodilatory properties of beta-agonists, this physiological process could explain the increased asthma morbidity associated with stress that has been indicated in children with asthma (Sandel & Wright, 2006; Wright, 2011). This mechanism may be particularly significant in children with problematic severe asthma because resistance to asthma therapy is a feature of problematic severe asthma.

A meta-analytic review suggested that parenting stress is higher in caregivers of children with any chronic illness than in caregivers of healthy children (Cousino & Hazen, 2013). However, findings about the occurrence of parenting stress in caregivers of children with asthma are equivocal. Most evidence indicates that parenting stress in children with asthma is in the normal range compared to healthy controls and norm reference groups (Markson & Fiese, 2000; Caffrey-Craig, 2005; DeMore, Adams, & Wilson, 2005). Only in one study parenting stress was found to be slightly higher in parents of children with asthma as compared to parents of children with cystic fibrosis or cancer (Hullmann et al., 2010). The authors suggested that higher parenting stress may follow from the daily demands that are placed on parents from children with asthma and diabetes. This could imply that parenting stress might be relatively high for parents of children with problematic severe asthma. However, the one study on controller medication in children with persistent asthma observed normal parenting stress levels (Walker, Papadopoulos, & Hussein, 2007).

An innovative aspect of our study is that it uniquely focuses on problematic severe asthma, which is characterized by longer periods of unstable asthma, lower forced expiratory flow in 1 s (FEV_1_), higher dose of inhaled steroids and more severe airway obstruction at the time of referral to a specialist (Hedlin et al., 2010). Since problematic severe asthma comprises difficult asthma and therapy resistant asthma, it represents the most severe group of asthmatic children. Parenting stress in caregivers of children with problematic severe asthma has not been investigated. Knowing the severity of parenting stress in this group is important because parenting stress may play a role in perseverance of asthma. Moreover, if the hypothesis of high parenting stress is also not verified in parents of children with problematic severe asthma (PSA), it indicates that parenting stress is likely not a factor playing a role in the persistence of PSA. We expected that parenting stress would be high in this group considering the great concern and responsibility of parents for children with problematic severe asthma.

Regarding the interplay between parenting stress and asthma severity, some previous studies in asthma did indicate that greater parenting stress was associated with higher asthma severity (Kaugars, Klinnert, & Bender, 2004; Sandel & Wright, 2006), poorer illness management, poorer adherence to medication (Celano, Klinnert, Holsey, & McQuaid, 2011), and poor house dust mite control (Joseph, Adams, Cottrell, Hogan, & Wilson, 2003). Moreover, it has been indicated that negative life events increase the risk of children’s asthma attacks (Sandberg et al., 2000) and that caregiver stress predicts wheeze in early childhood (Wright, Cohen, Carey, Weiss, & Gold, 2002). However, other studies concluded that age of asthma onset, peak flow variability, and illness severity were unrelated to general parenting stress (Caffrey-Craig, 2005; Chiou & Hsieh, 2008) or even that greater parenting stress was correlated with greater medication (inhaler) adherence (DeMore et al., 2005). Thus, studies of the association between asthma severity in children and parenting stress have not yielded uniform results.

Our previous study observed that, as compared to healthy reference groups, children and adolescents with problematic severe asthma had more internalizing behavioral problems such as being withdrawn/depressed and more severe somatic complaints (Verkleij et al., 2011). Parental stress is a potential determinant of behavioral problems in the children as indicated by a study that observed a correlation between a negative family emotional climate and child internalizing symptoms (Lim, Wood, & Miller, 2008). As yet, however, in children with problematic severe asthma, the association of parenting stress with the child’s behavioral problems has not been studied.

The aim of the current study was to examine the presence of parenting stress as related to the parent’s own functioning and the functioning of the child in a sample of children with problematic severe asthma, and to examine the relation of parenting stress with disease severity and behavioral problems in their children. We hypothesized that the level of parenting stress is higher than in the general population and that parenting stress is associated with asthma severity and behavioral problems in their children.

## Methods

### Study Population

A cross-sectional study examined Dutch children and adolescents with asthma before the start of inpatient treatment in a high altitude asthma clinic with a hypo-allergenic environment in Switzerland, the *Merem Netherlands Asthma Center Davos (NAD),* or an asthma clinic at sea level in the Netherlands, the *Merem Asthma Center Heideheuvel (ACH)*. These clinics were chosen because both are referral centers for children and adolescents with problematic severe asthma (Hedlin et al., 2010; Lødrup Carlsen et al., 2011). As described in detail previously (Van de Griendt, Verkleij, Douwes, van Aalderen, & Geenen, 2014), patients are admitted to the clinics when they have persistent symptoms as certified by a specialized pulmonologist (according to Global Initiative for Asthma 2012 criteria) despite treatment in step 3 (i.e. double dose of inhaled steroids and/or need for additional long acting beta-2 agonists or leukotriene receptor antagonist) or higher (Global Strategy for Asthma Management and Prevention, Global Initiative for Asthma [GINA], 2012). Although a reduction in forced expiratory volume in 1 s (FEV_1_) supports the diagnosis of problematic severe asthma, many children with severe, therapy-resistant, asthma have normal spirometry in asymptomatic periods (Lødrup Carlsen et al., 2011). Allergy for one or more inhaled allergens and eczema or other presentations of the atopic syndrome are commonly present. The reason for referral to one of the centers by the local or academic pediatric pulmonologist is the instability of the asthma and comorbidity such as allergic rhinitis, eczema, dysfunctional breathing and psychosocial problems. This needs an intensive multidisciplinary approach to therapeutically target these problems. A standard diagnostic program is performed with somatic and psychosocial investigations (Verkleij et al., 2013). Both clinics provide highly integrated multidisciplinary treatment programs of 1–3 months duration. The children participate in a group psycho-educational asthma program that aims to increase knowledge, technical skills (inhalation technique), and coping strategies. Moreover, they have individual therapeutic contacts with a pediatric pulmonologist or pediatrician, pulmonary nurse, physical and sports therapist, pedagogical worker, psychologist, and social worker. The parents participate in an educational program.

Several factors are unique to the treatment in Davos, Switzerland in contrast to children treated at sea level in Hilversum, the Netherlands. The children in Davos live in a hypo-allergenic environment due to a lower concentration of pollen and almost complete absence of house dust mite (Spieksma, Zuidema, & Leupen, 1971). The patients in Switzerland live separated from their family and their own social network. They all remain there for the whole treatment period (including the weekends). In contrast, the children that are treated in the Netherlands are at home every weekend.

**Fig. 1:**
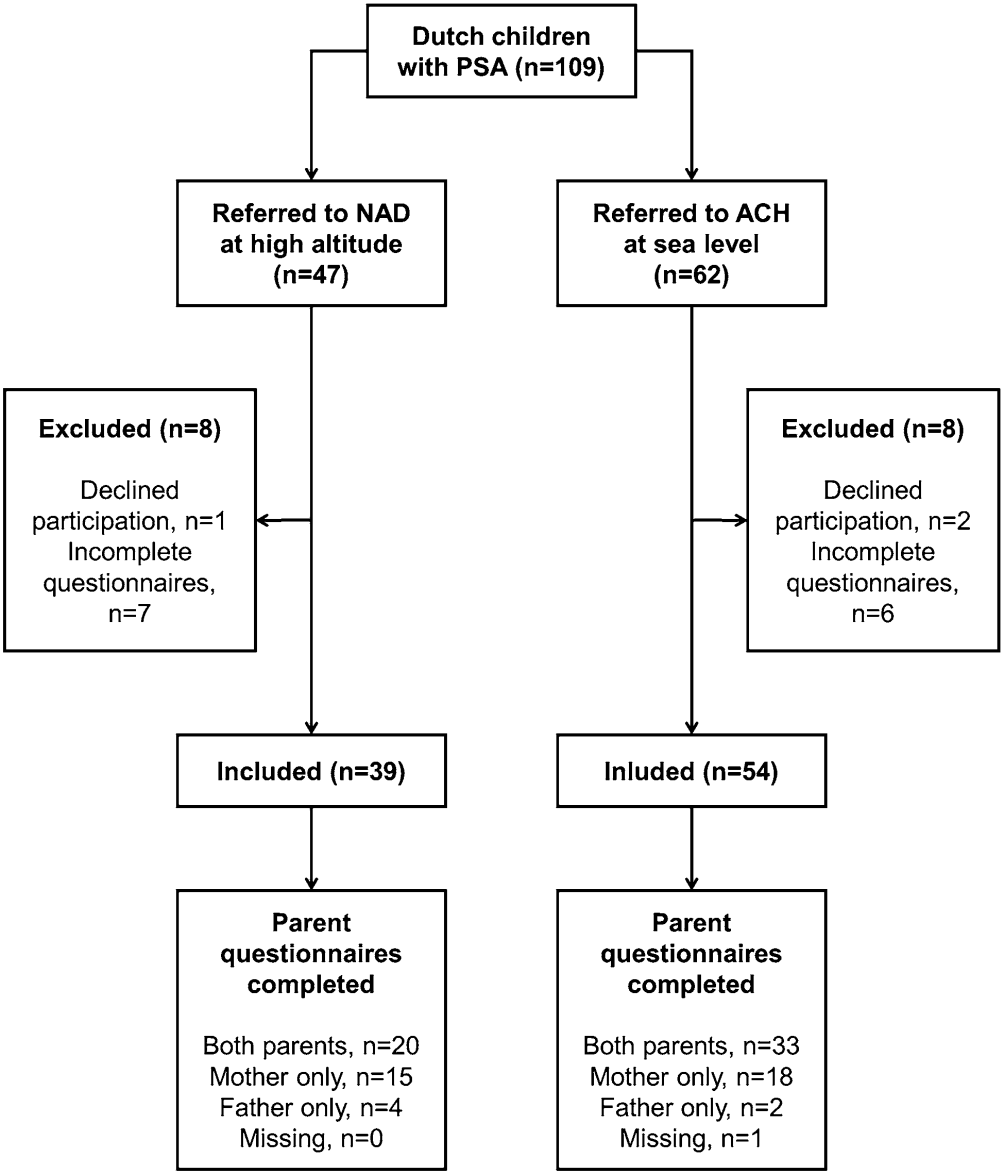
Flowchart

Figure 1 shows the flow chart of the study. Of the 93 children in this study, 39 were from NAD and 54 from ACH. In the NAD population, 47 children were eligible for inclusion, 39 (83 %) were included; one child did not provide informed consent, seven did not return all questionnaires. Of the 62 children of ACH, 54 children were included (87 %); two children did not provide informed consent and 6 did not return all questionnaires. Eighty-six mothers and 59 fathers of the 93 children completed the PSI/NOSI-questionnaire; with 57 complete dyads.

From 2010 to 2012 all children aged 7–18 years who were referred to one of the two tertiary clinics because of problematic severe asthma were invited to participate in the study. The medical ethics committee of the Amsterdam Medical Center (AMC), Amsterdam, the Netherlands, approved the study. All parents and children aged 12 and older provided written consent, children younger than 12 provided oral assent.

### Procedure

Two weeks before the start of clinical treatment in one of the specialized asthma clinics, the patients and parents received questionnaires at their homes. Medical history and physical examination were performed on the day of arrival by the pediatrician. Medical history included atopic symptoms, exercise intolerance, medication, reliever therapy and adherence as derived from the clinical interview. Pulmonary function testing was performed.

### Instruments

#### Descriptive Variables

Demographic variables that were measured in the children were gender and age. For both parents, age, country of birth, relational status, number of children, chronic illness and education level were measured.

#### Parenting Stress-Index (PSI/NOSI)

The Parenting Stress Index (PSI/NOSI) assesses the multidimensionality of parenting stress including such aspects as emotional distress in the parenting role, the parent’s ability to cope with the task of parenting and the parents’ perceptions of the child’s demands (Abidin, 1990). We used the Dutch adapted version of the PSI, the “Nijmeegse Ouderlijke Stress Index” (NOSI; De Brock, Vermulst, Gerris, & Abidin, 1992), which is named PSI/NOSI in this paper. This self-report inventory measures parenting stress using 123 items divided into two major domains of 13 subscales. The “parent domain” that refers to perceived stress regarding family factors includes seven subscales: Sense of Competence (e.g., “Parenting this child is more difficult for me than I expected”), Restriction of Role (e.g., “I often get the feeling that my child’s needs control my life”), Attachment (e.g., “I find it difficult to understand what my child wants or needs from me”), Depression (e.g., “Sometimes, I am so tired in the morning, that I don’t feel like getting up to take care of my children”), Parent’s Health (e.g., “I had more health complaints in the past 6 months than normally”), Social Isolation (e.g., “I feel alone and have no friends”), and Relationship with Spouse (e.g., “My partner and I often disagree on how to manage our child”). The “child domain” that refers to stress evoked by their child’s behavior and emotions contains six subscales: Adaptability (e.g., “My child gets upset in unexpected situations”), Acceptability (e.g., “It is difficult for me to accept my child as it is”), Demandingness (e.g., “Compared to other children, my child demands more of me”), Mood (e.g., “My child is often bad-tempered”), Distractibility- Hyperactivity (e.g., “It is very difficult to my child to sit still for a period”), and Reinforcement to the Child (e.g., “I often get the feeling that my child does not like me”). The items are scored on a six-point Likert-scale ranging from “totally disagree” = 1 to “totally agree” = 6.

The internal consistency reliability of the total parenting stress score in the clinical and non-clinical population is good (Cronbach’s alpha = .94; De Brock et al., 1992). Concurrent validity ranges from “satisfactory” to “good” and discriminant validity is considered adequate (De Brock et al., 1992). Cronbach’s alpha in our study sample showed good internal consistency reliability for the total parenting stress score in mothers (α = .92) and fathers (α = .89.), the parent domain mothers α = .89, parent domain fathers α = .87, child domain mothers α = .89, and child domain fathers α = .91. Cronbach’s alpha for the subscales ranged from α = .84 (“Depression” of parent domain mothers) to α = .91 (“Reinforcement to the child” of child domain fathers). In the analyses, we used the domain scores (parent and child domain) for fathers and mothers separately.

*The life events scale* is part of the Dutch version of the PSI/NOSI. It consists of 40 life events. Respondents answered with “yes” or “no” to the question whether a life event occurred within the family during the past 12 months, such as divorce, discharge, debts, miscarriage, and bereavement. There was no psychometric evaluation available. The total sum score of life events was used in analysis (theoretical range 0–40).

#### Parental Report: The Child Behavior Checklist (CBCL)

The CBCL is a standardized questionnaire that uses ratings by parents or caregivers to assess emotional and behavioral problems of children and adolescents (Achenbach & Rescorla, 2007). Parents of the children and adolescents filled out the Dutch version of the CBCL (6–18 years; Achenbach & Rescorla, 2007). The CBCL consists of 120 items to which participants respond on a 3-point Likert scale comprising 0 = “Not at all”, 1 = “Somewhat or sometimes”, and 2 = “Obvious or Often.” Results of the CBCL are expressed in a global score (120 items, range 0–240) and in scores for the domains internalizing (32 items, range 0–64) and externalizing (35 items, range 0–70) behavioral problems. Internalizing behavioral problems include the syndrome domains anxious/depressed, withdrawn/depressed and somatic complaints. Externalizing behavioral problems include rule-breaking behavior and aggressive behavior. Three other syndrome domains are not part of the global scores: social problems, thought problems, and attention problems.

In all analyses CBCL *T*- scores were used. Higher scores indicate more behavioral problems. A *T*-score of 63 (90th percentile in the norm population) demarcates the clinical range, which is an indication that a child has clinically relevant symptoms and might need professional help. The Dutch version CBCL showed adequate psychometric values and good reliability (Achenbach & Rescorla, 2007). Cronbach’s alpha coefficients in our study sample were .87 for the total CBCL score (eight scales), .74 for internalizing behavioral problems (three scales), and .57 for externalizing behavioral problems (two scales).

#### Children’s Self-report: Asthma Control

The childhood asthma control test (ACT; Childhood Asthma Control Test, 2008; Liu et al., 2007) assesses the control of asthma at the moment of measurement, e.g., “How is your asthma today?”, “How much of a problem is your asthma when you run, exercise, or play sports?”, “Do you cough because of your asthma?”, and “Do you wake up during the night because of your asthma?”. The child completes these items using a 4-point response scale, with lower scores indicating poorer control. The summated total score ranges from 0 to 12.

#### Lung Function

Pulmonary function testing was performed using the Masterscreen (Jaeger^®^, CareFusion Corporation). Children performed a manoeuvre of forced exhalation in a mouthpiece that was connected to a spirometer. According to the standardized protocol at least 3 technically correct manoeuvres had to be performed. Short or long acting β_2_-adrenergic agonists were stopped 12 h before testing. The lung function parameter that was obtained and evaluated was forced expiratory volume in 1 s (FEV_1_). Lung function measures were plotted in a reference set and expressed as percentage of the predicted (% predicted) value at this age, weight and height. In children, in between asthma attacks, even in severe asthma, lung function is often within a normal range of 80 to 120 % predicted. Sequential measurements are a valuable tool to evaluate change in bronchoconstriction in individual children with asthma; however, FEV_1_ measures are not a valid variable to indicate individual differences. Therefore, no correlations between FEV_1_ and parenting stress were computed.

Airway inflammation was measured with the NIOX^®^Flex Nitric Oxide Monitoring System (Aerocrine AB, Solna, Sweden) using the fractional concentration of exhaled nitric oxide (FeNO) according to the American Thoracic Society (ATS) and European Respiratory Society (ERS) guidelines (ATS/ERS, 2005). Children exhaled quietly in a mouthpiece that was connected to an analyzer. A higher fraction of exhaled nitric oxide denotes more eosinophilic inflammation (Dweik et al., 2011). Eosinophils are blood cells that play an important role in the inflammatory response that finally leads to bronchoconstriction in allergic asthma. Normal values in children range from 10 to 25 parts per billion.

### Statistical Analysis

Statistical analyses were performed with SPSS 20 and Mplus 6.1 (Muthén & Muthén, 2007). The score distributions were checked for outliers and normality.

PSI/NOSI scores were categorized in classes derived from a Dutch non-clinical reference population (De Brock et al., 1992). In this non-clinical population of 161 mothers and 84 fathers, 35, 30, and 35 % of the parents were classified to have lower than average [PSI/NOSI cut score: ≤226 for mothers and ≤214 for fathers], average [≤292 for mothers and ≤270 for fathers] and higher than average [≥293 for mothers and ≥271 for fathers] levels of PSI/NOSI parenting stress scores. Higher scores correspond to more parenting stress.

Moreover, Cohen’s effect size estimates (*d*) were calculated for each parent using the mean and standard deviation of this non-clinical population (De Brock et al., 1992) as reference values. The interpretation of these effect sizes is as follows: 0.2 ≤ *d* < 0.5 indicates a small effect, 0.5 ≤ *d* < 0.8 a medium effect, and *d* ≥ 0.8 a large effect (Cohen, 1977). Since some effect sizes had skewed score distributions, deviations from the non-clinical reference groups were examined with the nonparametric one-sample Wilcoxon signed rank test. Pearson correlations were computed to reflect the univariate associations between the variables of interest.

Path analysis or structural equation modelling (SEM) was used to examine whether the parent variables, life events, and the child variables, internalizing behavioral problems, externalizing behavioral problems, and FeNO, were associated with the four measures of parenting stress, i.e., with mothers’ and fathers’ responses to PSI/NOSI parent domain items and child domain items. The advantage of SEM is that it accounts for the shared variance resulting from mothers and fathers reporting on the same child. A disadvantage of SEM is that it requires relatively large sample sizes.

To deal with this issue, the results were tested in two steps, starting with the usual approach using the maximum likelihood estimator robust to non-normality (MLR). The MLR estimation results in the same parameter estimates as maximum likelihood estimation (ML) but Chi Square is corrected for non-normality (Hox, Maas, & Brinkhuis, 2010). To deal with missing observations of either the mother or the father full information maximum likelihood (FIML) was used to estimate these missing values, which allows the use of all available information.

In the next step, the same model was re-analysed using the Bayesian estimator. The Bayesian approach is more appropriate to deal with small sample sizes and the underlying non-normal distribution of the data (Van de Schoot et al., 2014; Van de Schoot, Broere, Perryck, Zondervan-Zwijnenburg, & Van Loey, 2015). Model fit was evaluated using the Chi Square statistic and three model fit indices: the Tucker Lewis Index (TLI), the comparative fit index (CFI), and the root mean square error of approximation (RMSEA; Kline, 2011). Conventional guidelines suggest that model fit indices TLI and CFI between .80 and .90 and RMSEA between .05 and .08 represent a moderately fitting model, whereas TLI and CFI values >.90 and RMSEA <.05 represent good model fit. Chi Square should preferably be non-significant (Kline, 2011).

Re-estimation of the model with the Bayesian estimator provides posterior estimates, the posterior *SD* and the 95 % asymmetric credibility intervals of this model. Credibility intervals, the Bayesian counterpart of the confidence intervals, offer information about the robustness of the findings. We used the default prior settings of the software (i.e., uninformative priors). The Bayesian estimator uses multiple chains and iterations to attain a stable posterior distribution (Van de Schoot et al., 2014). The chain can start at any arbitrarily chosen point and iterates many times until it appears as coming from the target distribution. In this study, the starting values were based on the results from the MLR model, then 20,000 iterations were used with 15 chains to decide if the chains had reached their stationary distribution or desired posterior. The first 50 % of the iterations were ignored (i.e. burn-in phase) in the computation of the posterior to avoid any influence of the arbitrary chosen starting values. Convergence was assessed using the potential scale reduction (PSR) convergence criterion (Gelman & Rubin, 1992). This criterion compares the variances within each chain and the variance between chains. Large deviations between the variances are indicative for non-convergence. The PSR was estimated to be <1.02 of 50 % of the total number of iterations. This shows good convergence properties. Also all trace-plots were visually inspected for non-convergence. Trace-plots are useful in assessing convergence as it shows if the chain is mixing well and if the chain has reached stationarity when there is a relatively stable mean and variance. The trace-plots in this study were all doing well. The model was re-analyzed using twice as many iterations and the posterior estimates did not differ indicating convergence was reached.

## Results

### Characteristics of the 93 Children and Adolescents

Table 1 shows the characteristics of the 93 children and adolescents with a complete data set. The mean FEV_1_ measurement was in the normal range. Mean FeNO was slightly elevated (normal values range from 10–25 ppb). Overall, the mean for CBCL *T*-scores fell in the normal range, but for this sample of children with asthma: 29.4 % scored in the borderline clinical significant range on the total problem score (*T*-score ≥ 60; 84th percentile); 39.1 % in the borderline clinical significant range for internalizing behavioral problems; and for externalizing behavioral problems, 16.3 % fell within that range. On average, ACT scores reflected poor control of asthma.

**Table 1:** Characteristics of the 93 children and adolescents

	Children and adolescents *n* = 93	
Female, number (%)	48	(51.6 %)
Age of child, mean (*SD*), range years	13.4	(2.7) 7–18
Behavioral problems (CBCL), mean *T*-scores (*SD*)^a^		
Total score	53.7	(9.7)
Internalizing	57.1	(9.7)
Externalizing	48.5	(10.1)
Lung function		
FEV_1_(*SD*)^b^	100.9	(15.5)
FeNO (*SD*)^c^	28.5	(25.6)
Control of asthma (ACT)^d^		
Total child score (*SD*)	6.1	(2.5)

^a^CBCL = Child Behavior Checklist (*T*-score ≥ 63; 90th percentile = clinical significant range; a higher score reflects more problems)

^b^FEV_1_ (forced expiratory volume in 1 s) is expressed as percent of predicted (% pred)

^c^FeNO (fractional concentration of exhaled nitric oxide) expressed as parts per billion (ppb; normal range 10–25 ppb; a higher value corresponds with more eosinophilic inflammation)

^d^ACT = Childhood Asthma Control Test (range 0–12; a higher score reflects better control)

### Characteristics of the 145 Parents

Table 2 shows the characteristics of the 145 fathers and mothers of the 93 patients before treatment in one of the two treatment centers. The number of parents with a chronic illness was high; 39.8 % of the children had at least one parent with a chronic illness, mostly a lung disease. Of the life events, events within the family (birth, divorce, death, and so on) were most frequently reported by the fathers and mothers.

**Table 2:** Characteristics of the parents of the 93 children and adolescents

Total group fathers and mothers	*n* **=** 145
Relational status (%)	
Married and living together	76.3 %
Living apart together	2.2 %
Divorced and living apart, single	21.6 %
Widowed	0 %
Mean number of children per family (*SD*)	2.43 (.88)
Mean number of children per age category (*SD*)	
Age <4	.08 (.30)
Age 4–12	.82 (.88)
Age 12–18	1.25 (.87)
Age >18	.27 (.71)
Child with parent with chronic illness, *n* (%)	37 (39.8 %)

	Mothers, *n* = 86	Fathers, *n* = 59
Parent with chronic illness, *n* (%)	26 (30.2 %)	18 (30.5 %)
Lung disease*, n*	15	12
Rheumatic disease, *n*	3	0
Diabetes, *n*	1	0
Cardiovascular disease, *n*	1	2
Psychiatric or psychological, *n*	1	0
Other disease, *n*	7	5
Age, mean (*SD*)	42.6 (4.6)	45.0 (5.5)
Country of birth		
Netherlands/unknown/other country (*n*)	77/1/8	56/0/3
Education level^a^		
Low/middle/high (*n*)	14/53/19	8/29/22
Life events^b^, mean (*SD*) range	2.2 (1.9) 0–7	2.0 (2.0) 0–8

^a^Education level, “Low”: Primary school or lower vocational secondary education, “Middle”: intermediate general secondary education or intermediate vocational education, and “High”: higher general secondary education, higher vocational education, or university education

^b^The life events scale included 40 life events

### Parenting Stress Index (PSI/NOSI)

Using the categories derived from a non-clinical population (De Brock et al., 1992), more than half of the fathers and mothers of our sample reported parenting stress scores reflecting lower than average levels of parenting stress (Table 3), while 15 to 20 % obtained higher than average levels of parenting stress. In Table 4, columns 2 and 10 show the median of effect size deviation scores of the parents on parenting stress as compared to the reference scores of a non-clinical population of 161 mothers and 84 fathers (De Brock et al., 1992). The scores (*d*) reflect the difference in standard deviation units between the observed scores of the parents of children with asthma (our sample) and the parents of the non-clinical population, and thus are Cohen’s *d* effect sizes. Overall, the parents of children and adolescents with asthma reported less parenting stress than the non-clinical reference group: most effect sizes were medium to large. On the total PSI/NOSI-score, mothers showed a large deviation (*d* = 0.8, *p* < .001) and fathers a medium deviation (0.5 ≤ *d* < 0.8, *p* < .001) from the non-clinical reference group.

**Table 3:** Percentages of mothers and fathers of children with asthma reporting lower than average, average, and higher than average levels of parenting stress as compared to parents from a non-clinical population

	Percentages		
PSI scale	Lower than average	Average	Higher than average
Mothers (*n* = 86)			
Parent domain total score	55.8	30.2	14.0
Child domain total score	54.7	29.1	16.3
Total score	58.1	27.9	14.0
Fathers (*n* = 59)			
Parent domain total score	52.5	32.2	15.3
Child domain total score	55.9	23.7	20.3
Total score	54.2	25.4	20.3
Non-clinical population (*n* = 245)			
Total score	35	30	35

*PSI* Parenting stress index

**Table 4:** Medians of effect sizes (*d*-scores) of the parenting stress index (PSI/NOSI) of mothers and fathers of the current sample compared to a non-clinical sample of the original validation article (De Brock et al., 1992) and the mean scores of the current sample, the non-clinical and clinical samples of the original validation article, and samples of children with and without enuresis (De Bruyne et al., 2009) and with normal weight and overweight (Moens et al., 2009)

PSI	Mothers *n* = 86							
	Median effect size deviation	Current sample	Non-clinical sample^a^	Clinical sample^a^	Children with enuresis^b^	Children without enuresis^b^	Normal weight group^c^	Overweight group^c^
Parent domain								
Total score	−.71**	98.2 (30.8)	121.0 (34.9)	151.8 (42.6)	125.4 (45.1)	102.3 (35.4)	113.6 (34.9)	125.7 (40.1)
Sense of competence	−1.1**	21.4 (6.7)	29.4 (9.1)	38.1 (11.2)	27.4 (11.1)	20.8 (9.3)		
Restriction of role	−.40*	13.4 (6.1)	14.3 (5.8)	16.9 (7.2)	17.1 (7.6)	13.6 (6.1)		
Attachment	−1.0**	9.4 (3.0)	12.3 (4.3)	16.5 (5.3)	15.0 (3.5)	13.3 (2.8)		
Depression	−.81**	19.5 (7.6)	26.8 (9.6)	33.4 (11.1)	25.2 (10.7)	20.3 (8.7)		
Parents health	−.52*	11.9 (5.6)	13.6 (5.0)	15.8 (6.2)	14.2 (6.7)	11.5 (5.3)		
Social isolation	−.90**	9.1 (4.1)	10.8 (4.2)	11.8 (4.8)	11.6 (5.3)	9.3 (4.2)		
Relationship with spouse	−.22	13.4 (6.4)	13.5 (6.8)	19.0 (8.9)	15.0 (8.2)	13.5 (7.2)		
Child domain								
Total score	−.78**	126.1 (40.6)	145.3 (37.3)	201.1 (43.1)	152.5 (53.2)	119.2 (34.5)	144.6 (49.6)	151.9 (49.8)
Adaptability	−1.20**	25.1 (10.9)	32.3 (8.6)	42.2 (10.2)	30.8 (12.0)	26.2 (8.7)		
Acceptability	−.74**	18.9 (6.5)	22.6 (7.6)	32.6 (9.5)	23.3 (10.5)	16.9 (6.2)		
Demandingness	−.14	21.5 (8.5)	20.8 (7.3)	32.5 (8.0)	24.7 (11.6)	16.6 (7.5)		
Mood	−.75**	18.5 (9.0)	21.7 (7.6)	30.6 (9.0)	24.2 (9.2)	20.3 (6.4)		
Distractibility−Hyperactivity	−.46*	28.1 (9.5)	30.6 (11.0)	42.7 (11.5)	33.8 (13.0)	25.4 (9.4)		
Reinforcement to Parent	−.83**	14.03 (4.7)	17.3 (5.2)	20.7 (6.2)	15.9 (5.8)	13.8 (4.8)		
Total score	−.81**	224.3 (66.9)	266.5 (66.9)	353.2 (78.5)	277.9 (91.3)	221.5 (65.0)	258.3 (78.7)	277.6 (11.5)

PSI	Fathers *n* = 59					
	Median effect size deviation	Current sample	Non-clinical sample^a^	Clinical sample^a^	Children with enuresis^b^	Children without enuresis^b^
Parent domain						
Total score	−.79**	92.5 (28.7)	108. 1 (28.1)	128.0 (37.2)	110.1 (36.6)	92.2 (31.1)
Sense of competence	−.96**	20.8 (7.2)	25.9 (7.2)	32.3 (9.2)	23.5 (10.4)	18.0 (7.6)
Restriction of role	−.62*	12.2 (6.0)	13.1 (5.0)	16.0 (7.0)	16.1 (7.1)	12.2 (6.0)
Attachment	−1.1**	9.9 (3.4)	12.2 (3.7)	14.6 (5.5)	14.6 (3.5)	13.3 (2.7)
Depression	−.85**	17.7 (5.4)	21.6 (6.6)	25.5 (9.7)	19.8 (7.0)	17.8 (6.4)
Parents health	−.38*	10.1 (3.7)	11.4 (3.7)	13.1 (4.7)	11.3 (5.2)	9.6 (4.1)
Social isolation	−.72*	9.5 (4.5)	11.4 (4.7)	11.1 (5.2)	11.3 (4.8)	9.1 (4.0)
Relationship with spouse	−.27	12.2 (6.7)	12.2 (4.4)	14.4 (6.8)	13.6 (6.1)	12.3 (7.1)
Child domain						
Total score	−.87**	121.5 (39.1)	142. 2 (37.1)	186.2 (42.0)	141.1 (43.0)	111.1 (27.1)
Adaptability	−1.2**	25.0 (10.2)	32.4 (8.7)	41.5 (10.0)	28.3 (9.2)	24.5 (6.5)
Acceptability	−.46*	19.0 (6.7)	21.2 (7.0)	28.3 (9.3)	21.3 (9.0)	15.8 (4.7)
Demandingness	−.52	20.6 (8.8)	20.3 (6.4)	29.4 (9.7)	21.5 (9.5)	15.0 (6.0)
Mood	−1.0**	16.5 (7.4)	20.7 (6.7)	28.0 (7.8)	22.7 (7.1)	18.7 (5.2)
Distractibility−Hyperactivity	−.65**	25.6 (7.7)	31.0 (10.7)	40.5 (11.8)	32.1 (10.1)	24.6 (8.7)
Reinforcement to Parent	−.55*	14.8 (5.9)	16.6 (4.7)	18.5 (4.9)	15.2 (5.4)	12.6 (4.1)
Total score	−.67**	214.0 (64.6)	250.4 (60.7)	314.5 (72.1)	251.1 (74.9)	203.3 (54.3)

The effect size (*d*) reflects the magnitude of deviation from the non-clinical reference population in standard deviation units; a negative score indicates that the parents of children with asthma are judged to have less stress than the reference population. The *d* values have the following common effect sizes: values smaller than 0.2 reflect no deviation from the reference population; values between 0.2 and 0.5 reflect small deviations; values between 0.5 and 0.8 reflect medium deviations; and values greater than 0.8 reflect large deviations. The significance of these median effect size deviations from the non-clinical reference population were tested using one-sample Wilcoxon signed rank tests

The significance of differences between the mean parenting stress scores of the current sample and other samples is reported in the text

*SD*s associated with means are shown in parentheses

A significant score reflects that the number of parents with lower scores than the mean score of the reference population exceeds chance; *p < .05, **p < .001

^a^Reference population scores of the PSI/NOSI (De Brock et al., 1992)

^b^Population scores of 78 children with enuresis vs 110 without enuresis (De Bruyne et al., 2009), wherein parenting stress was measured with the PSI/NOSI

^c^Population scores of 100 children with overweight and 97 with normal weight (Moens et al., 2009), wherein parenting stress of mothers was measured with the PSI/NOSI

The deviation of the total score of parenting stress (parent domain) was medium for mothers and fathers (0.5 ≤ *d* < 0.8, *p* < .001). On the stress evoked by their child’s behavior and emotions (child domain), mothers showed a medium deviation (0.5 ≤ *d* < 0.8, *p* < .001) and fathers a large deviation (*d* ≥ 0.8, *p* < .001) from the non-clinical reference group.

On most subscales of the parent domain, both mothers and fathers showed a medium to large deviation from the reference comparison group in the direction of lower levels of stress, especially with respect to “Sense of Competence,” “Attachment,” “Depression,” “Parent’s Health,” and “Social Isolation.” On the child domain subscales, deviations were large for “reinforcement to parent” (mothers) and “mood” (fathers) and medium for “acceptability” (mothers), “distractibility-hyperactivity” (fathers) and “reinforcement to parent” (fathers).

Because our design lacked a matched control group and the parenting stress of our study group was unexpectedly low as compared to parenting stress in the non-clinical reference group, we decided to compare the parenting stress scores to scores of other clinical and non-clinical samples that used the Dutch version of the PSI/NOSI (Table 4). Compared to the Dutch reference scores of the clinical sample of the PSI/NOSI (mothers *n* = 68, fathers *n* = 40; De Brock et al., 1992), effect size deviations were even larger than the deviation from reference scores of the non-clinical population (see Table 4). Compared to a study sample of parents with children with enuresis (*n* = 78, aged 5–13 years) using the PSI/NOSI (De Bruyne et al., 2009), our study sample showed significantly less parenting stress in fathers and mothers on all domains (*p* < .001). The population of our study was comparable with the control group without enuresis (*n* = 110, aged 5–15 years) in this study (De Bruyne et al., 2009) on all domains in mothers: parent domain (*t* = −1.24, *p* = .22), child domain (*t* = 1.57, *p* = .12) and total score (*t* = .38, *p* = .70). The same was true for the fathers parent domain (*t* = .09, *p* = .93) and total score (*t* = 1.27, *p* = .21), while the fathers of the current sample scored significantly higher than the fathers of the control group without enuresis on the child domain (*t* = 2.04, *p* = .046). Compared with a cross-sectional study of 197 families with children with normal weight (*n* = 97) and overweight (*n* = 100) aged 6–14 years (Moens, Braet, Bosmans, & Rosseel, 2009), our study sample showed significantly less parenting stress in the mothers on all domains (*p* < .001).

### Path Analysis Examining Variables Associated with Parenting Stress

In the first step of path analysis, associations of parental life events, child internalizing behavioral and externalizing behavioral problems, and FeNO with parenting stress reported by both the mother and father were examined. The model included 93 families of which 57 reports from both mother and father were available. The model using MLR estimation showed the five variables to be statistically significantly related to parenting stress and provided a moderate to good fit (*χ*^2^ = 6.99, *df* = 4, *p* = .14; RMSEA = .09; CFI = .99; TLI = .91). The model using Bayes estimation showed the same results excluding the associations of externalising problem behavior with maternal parenting stress and of FeNO with the child domain of parenting stress as reported by mothers that did no longer reach statistical significance. As the credibility interval did not include zero in any of the estimated parameters, it can be concluded that the results reflect robust findings.

**Fig. 2:**
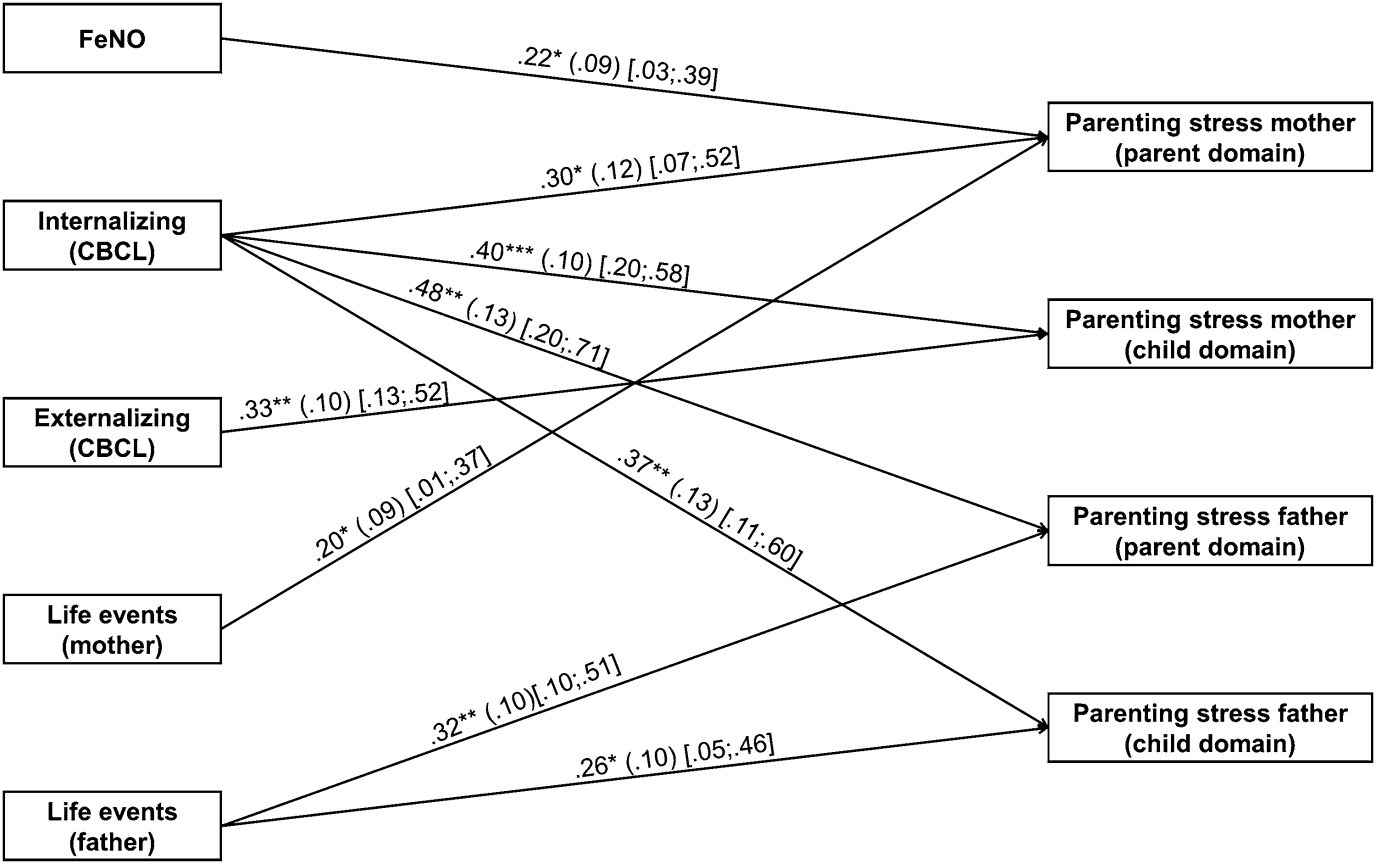
Associations of airway inflammation (FeNO) of the child, internalizing and externaling behavioral problems of the child, and life events of the parents with parenting stress. The posterior estimates, the posterior *SD* and the 95 % asymmetric credibility intervals of the model are given. *p < .05, **p < .01, ***p < .001

The Bayesian outcomes are presented in Fig. 2, which shows paths that were significant using the Bayesian analysis that adjusts for small sample size. Attached to each path are four associated numerical values: the posterior estimate with significance level; the *SD* linked to that posterior estimate; and the lower and upper 95 % asymmetric credibility intervals for that path in this model. For example, the uppermost path in Fig. 2 shows that, the path from FeNO to Parenting stress of the mother (parent domain) was significant at the .05 level; the posterior estimate = .22, with *SD* = .06, and 95 % credibility interval ranging from .03 to .39. For the model as a whole, the 95 % credibility interval for the difference between the observed and the replicated Chi square values was estimated to be −23.98 to 38.46, and the posterior predictive *p* value was .34, both indicating a good posterior-predictive model fit. A higher score on internalizing behavioral problems of the child was associated with more parenting stress in all parenting stress domains. More externalizing behavioral problems were associated with higher parenting stress scores in the child domain reported by the mother. Regarding lung function assessment, the FeNO indication of greater inflammatory activity in the child was associated with higher maternal parenting stress. The association between FeNO and the child domain as reported by the mother fell just short of significance in the model when other relationships were taken into account, and yielded the following estimate: .16 (.08) [−0.00; .33], *p* = .052. Finally, life events of the parents were associated with higher parenting stress scores in the mother domain whereas in fathers, it was associated with both the parent and child domain.

The Pearson correlations of the variables that were tested in path analysis are shown in Table 5. The significance of the univariate correlations (not corrected for other variables) largely reflect the adjusted estimates as calculated in the path analysis.

**Table 5:** Univariate Pearson correlations (and *p*-values) between the variables

	Internalizing behavioral problems	Externalizing behavioral problems	FeNO (ppd)	Life events
Mothers’ observation				
Parent domain	.47 (*p* < .001)	.43 (*p* < .001)	.20 (*p* = .06)	.29 (*p* = .007)
Child domain	.62 (*p* < .001)	.61 (*p* < .001)	.13 (*p* = .23)	.28 (*p* = .009)
Fathers’ observation				
Parent domain	.40 (*p* = .002)	.13 (*p* = .34)	.04 (*p* = .78)	.28 (*p* = .03)
Child domain	.51 (*p* < .001)	.45 (*p* < .001)	.03 (*p* = .84)	.27 (*p* = .04)

*FeNO* fractional concentration of exhaled nitric oxide, *ppb* parts per billion

## Discussion

This study examined parenting stress in parents of children with problematic severe asthma and the association of parenting stress with behavioral problems and disease severity in their children. To our knowledge, this is the first study that observed a significant association between parenting stress and airway inflammation in this group of children beyond the well-established association between parenting stress and behavioral problems of the child. Moreover, the comparison with samples from previous research suggested that both in mothers and fathers, parenting stress scores were on average low showing a medium to large deviation from a non-clinical reference group.

In accordance with our hypothesis, higher levels of parenting stress were associated with the observation of more behavioral problems in their children. This is in agreement with previous studies of children with asthma (Wood et al., 2008; Miller et al., 2009; Lim et al., 2008), cystic fibrosis (Goldberg et al., 1997) and other chronic diseases (cancer, arthritis, cystic fibrosis, diabetes and sickle cell disease) as indicated in a recent systematic review (Cousino & Hazen, 2013). Higher levels of parenting stress have been found to be related to lower psychosocial well-being (Majnemer, Shevell, Rosenbaum, Law, & Poulin, 2007) and more externalizing problems (Friedman, Holmbeck, Jandasek, Zukerman, & Abad, 2004), internalizing problems (Lewin et al., 2005) and depressive symptoms (Mullins et al., 2007) in the child. Our cross-sectional data do not establish causality. Likely the relationship between parenting stress and child behavioral problems is bidirectional, which can lead to an upward cycle that has negative consequences for both parent and child, i.e., higher levels of parental stress may increase a child’s behavioral problems, and the child’s increased behavioral problems may increase parental stress (Baker et al., 2003; Jones & Prinz, 2005). It is in agreement with open systems models that parenting stress and the child’s behavior affect one another (Bruzzese, Unikel, Gallagher, Evans, & Colland, 2008; Minuchin et al., 1975; Wood et al., 2008). The association between parenting stress and behavioral problems in the child indicates the importance of a family approach to deal with problems of both the parent and the child.

A main finding of our study is that parenting stress in mothers was associated with airway inflammation of their children as measured by FeNO, which confirms our hypothesized relation between parenting stress and asthma severity in the child with problematic severe asthma. Two possible interpretations of the correlation between parenting stress and FeNO exist. First, as psychosocial stressors may trigger the expression of asthma (Wright, 2011), parenting stress of the mother may lead to stress in the child that is a possible vulnerability factor for inflammation through neuro-endocrine and immune mechanisms. A second possible explanation is that recurrent episodes of inflammation are especially difficult to handle by the mother. It is a common finding that mothers as compared to fathers are more distressed by negative life events happening to their child, be it a disease or accident (Bakker, Van Loey, Van der Heijden, & Van Son, 2012; Nelson & Gold, 2012) and use other ways of coping (Olff, Langeland, Draijer, & Gersons, 2007). It is conceivable that particularly in mothers of children with problematic severe asthma, more frequent episodes of inflammation in the child lead to increased helplessness, negative emotions, and other aspects of stress. Longitudinal studies are needed to examine the directionality of the association between episodes of exacerbation in the child and parenting stress in mothers.

Our study lacked a matched control group of parents. Comparison with all available samples using the PSI/NOSI indicated that levels of parenting stress in parents of tertiary treated children with problematic severe asthma are low, suggesting that raising a child with problematic severe asthma does, in general, not necessarily lead to enhanced parenting stress. This low level of parenting stress is surprising, because parenting stress may depend –among other influences– on the child’s problems and caregiver demands (Raina et al., 2005), which are higher in our group than in the general population (Verkleij et al., 2011). Although results of previous studies were not uniform, our hypothesis, guided by the biobehavioral family model, was that parenting stress in our population would be high instead of low. Afterwards, it is possible to mention some complementary explanations that may explain the low levels of parenting stress observed in the current study. According to the stress-appraisal model, parenting stress is not only determined by the severity of stressors but also by one’s capability to deal with stressors (Lazarus & Folkmann, 1984). In general, this group of parents may have learned to cope well with the disease of their child and have grown accustomed to their way of living and caring for their child. However, this would not explain why parenting stress for parents in our sample was lower than in reference groups whose children were not suffering from severe chronic disease. One possible way to understand this unexpected finding is by turning to concepts that assume stressors can have positive as well as negative consequences, as described under such headings as positive reappraisal (Folkmann & Moskowitz, 2000), positive reinterpretation (Carver, Schreier, & Weintraub, 1989), and benefit finding or growth (Schwartz, 2003). Some parents described caring for a chronic disabled child as a “commitment” that gave their life content and meaning (Chesla, 1991) or the feeling of achieving a mission in life ( Bulger, Wandersman, & Goldman, 1993; Hatfield, 1992). Thus, besides being a possible source of stress, caring for a child with a chronic illness like asthma may be a means to discover and create meaning and purpose in life, or it may increase the bond between parents and between the parents and the child. These ad hoc speculations should be further investigated in research.

Besides the possible explanations mentioned in the previous paragraph, also the specific population and moment of measurement may have played a role. The children in this study were on average 13 years old. At this age, children may be better able to communicate their health problems and take care of themselves than children who are younger. Parents of younger children may report higher levels of parenting stress as found in studies of parents of children under the age of five with asthma symptoms; these parents reported feeling frightened, helpless, frustrated, uncertain, vulnerable, and worried by symptoms that seem never to end (Kieckhefer & Ratcliffe, 2000). Furthermore, the timing of the measurement, right before the start of treatment, when the child was already accepted for treatment at the clinic, could have influenced parenting stress. Given that these children have chronic problems that could not be effectively managed at more routine levels of care, one would assume that these parents have been distressed and searching for better, higher level of care for their sick child. Once the child has been admitted for the treatment specialized tertiary care program, but before treatment has actually begun, one might expect that these parents would experience a sense of relief and a boost in their morale and hopefulness that now, at last, their child will get the best treatment possible. This sense of relief could be reflected in lower ratings of parenting stress. Overall, considering the effect sizes of differences with references groups, our finding of low levels of parenting stress in parents of children with problematic severe asthma tentatively indicated that parenting stress is low in this group.

There are some limitations to our study. First, we did not compare the parenting stress levels of our sample to parenting stress levels of a control sample matched on relevant demographic characteristics. Instead, we used all available reference groups that used this parenting stress questionnaire (PSI). These samples were obtained at a different time using a different sampling frame. This methodology is weaker than being able to directly compare our group of parents to a control group, which may pose a challenge to our interpretation—even though a wide range of comparisons indicated low parenting stress scores in our group. Second, the parents and children participated on a voluntary basis after having been informed about the purpose of the study. It is unknown whether our sample was representative for the population of parents of clinically treated children and adolescents with problematic severe asthma. However, non-participation among parents—especially among mothers—was low. Third, socially desirable responding may have occurred as in a previous study showing that 22 % of parents of children with asthma were responding defensively (DeMore et al., 2005), i.e., parents’ responses may have been influenced by a tendency to minimize negative aspects of their family situation and/or parental behavior. Fourth, the findings generalize only to parents of tertiary treated children with problematic severe asthma. As none of the demographic variables such as education level of the parents were related to the outcome variable, it is unlikely that demographic variables such as social economic status explain the findings.

With respect to implications for research, our results partially support the notion that, parents having lower control of their child’s asthma, as revealed in the child’s higher level of airway inflammation, is a stress factor for parents; alternatively, perhaps, parenting stress can aggravate the child’s asthma. Moreover, the behavioral problems of their children may be both a source for and a consequence of parenting stress. Longitudinal research is needed to get more insight regarding the direction of and mechanisms behind these associations.

With respect to clinical implications, high levels of parenting stress can lead to harsh or withdrawn parenting with consequences for child development (Deater-Deckard & Petrill, 2004). Professionals should be alert to parenting stress and behavioral problems in the child. When observing psychological problems in the parents or child, professionals can help to provide appropriate support in the relevant domains to reduce the primary sources of stress and improve well-being of the whole family (Markson & Fiese, 2000). Interventions aimed at empowerment and improving parenting skills of parents will benefit their chronically ill child (Hatzmann, Heymans, Ferrer-i-Carbonell, Praag, & Grootenhuis, 2008) in terms of a reduction of behavioral problems and asthma severity. Most important, our study indicates that care in this group involves a focus on all possible sources of problems, i.e., disease exacerbations and behavioral problems in the child as well as parenting stress.
